# ZnO nanoparticles act as supportive therapy in DSS-induced ulcerative colitis in mice by maintaining gut homeostasis and activating Nrf2 signaling

**DOI:** 10.1038/srep43126

**Published:** 2017-02-24

**Authors:** Jinquan Li, Hanqing Chen, Bing Wang, Chengxu Cai, Xu Yang, Zhifang Chai, Weiyue Feng

**Affiliations:** 1 CAS Key Laboratory for Biomedical Effects of Nanomaterials and Nanosafety, Institute of High Energy Physics, Chinese Academy of Sciences (CAS), Beijing 100049, China; 2 Section of Environmental Biomedicine, Hubei Key Laboratory of Genetic Regulation and Integrative Biology, College of Life Sciences, Central China Normal University, Wuhan, 430079, China; 3 National Center for Nanoscience and Technology, Beijing, 100190, China

## Abstract

Inflammatory bowel diseases (IBD) are widespread inflammatory diseases that cause debilitating health problems including cancer. In this study, we show that ZnO nanoparticle (ZnONP) treatment has markedly dose-dependent effects on the remission of dextran sulfate sodium (DSS)-induced ulcerative colitis in mice. We demonstrate the mechanism involves the antioxidant and anti-inflammatory abilities of ZnONPs to suppress ROS and malondialdehyde (MDA) production; increase GSH level; suppress proinflammatory cytokines IL-1β and TNF-α and myeloperoxidase (MPO). The ZnONP treatment is able to activate the Nrf2 pathway in the cellular antioxidant defense system. The novel finding is that ZnONP combined with mesalazine (5-ASA) can enhance the therapeutic efficacy of 5-ASA in the treatment of DSS-induced colitis. Lastly, we found that ZnONP treatment can restore the changes in special colonic bacteria of DSS-mice while the drug 5-ASA cannot. These results indicate that ZnONPs can act as a medical additive for the therapy of IBD.

Inflammatory bowel disease (IBD) is an idiopathic disease primarily includes two types of chronic intestinal disorders: ulcerative colitis (UC) and Crohn’s disease (CD)^1^ which affect approximately 1.4 million Americans with an accidence of 29.6 per 100,000^1^,^2^,^3^,^4^. The clinical manifestations of IBD are characterized by weight loss, colonic mucosal ulceration and diarrhea with blood or mucus^5^,^6^. So far, the exact etiology of IBD remains poorly understood. Oxidative stress is considered as a vital factor to initiate and propagate the development of IBD^7^,^8^. Recent studies have shown that the intestinal mucosa of UC, colon cancer and *Helicobacter pylori* infected tissues had 10- to 100-fold increased ROS and imbalance in antioxidant defense^9^. Ongoing efforts to widen the benefit-to-risk window of IBD therapy are urgently needed. The efforts are required on a number of complementary fronts. One potential therapeutic strategy is to effectively scavenge excessively generated ROS.

Nuclear factor-erythroid 2-related factor 2 (Nrf2) is a redox-sensitive transcription factor that plays an essential role in the promoter region of genes encoding antioxidant and/or detoxifying enzymes and stress-responsive proteins^10^,^11^. Activation of Nrf2 and antioxidant enzymes quinone oxidoreductase-1 (NQO-1) can attenuate inflammatory damage and neutralize ROS of cells/tissues from inflammatory injuries^12^,^13^. Recent studies have demonstrated that activation of Nrf2 can protect against dextran sulfate sodium (DSS)-induced colitis and inflammation-associated colorectal cancer by maintenance of intestinal integrity and regulation of proinflammatory cytokines^3^,^11^,^14^,^15^. *Nrf2* gene mutation might enhances the susceptibility and progression in DSS-induced colitis mice^3^,^15^. Activation of Nrf2 signaling with small molecule may represent a novel, safe and effective therapy in IBD patients.

Zinc (Zn) is one of the essential trace elements for several aspects of normal human development and health^16^,^17^. Growing evidence suggests that Zn involves in redox-regulated signaling and contributes to maintain the cell redox balance. which is associate with IBD^17^,^18^,^19^,^20^,^21^,^22^. Zinc supplementary therapy has been confirmed to effectively reduce the duration of acute and persistent diarrhea^19^. Zinc oxide (ZnO) is considered as a safe metal oxide compound and is approved by US Food and Drug Administration (FDA) for food and drug additive^23^. ZnO nanoparticles (ZnONPs) are commonly used in various applications including as drug carriers and fillings in medical materials and nutritional supplements^24^,^25^. Thus far, the potential therapeutic effect of ZnONPs on DSS-induced colitis is unknown.

In this study, we used biomedical approaches to investigate the therapeutic efficacy of ZnONPs and their protective mechanism in attenuating DSS-induced colitis model mice. We estimated the anti-oxidative and anti-inflammatory effects of ZnONPs in colitis tissues. Our data show that ZnONPs present more efficient therapeutic option for DSS-induced colitis than ZnO microparticle (ZnOMP) due to the more persistent Zn^2+^ release from ZnONPs. Moreover, we designed a novel therapeutic strategy of combining ZnONPs with antiulcer drug, mesalazine (5-aminosalicylic acid, 5-ASA), one of a commonly used drug for treating IBD, to the treatment of DSS-induced colitis in mice. The results obtained will have great potential to provide a new type of adjuvant agent for therapy of IBD.

## Results

### Characterization of ZnONP and ZnOMP suspension solution

**Figure 1:**
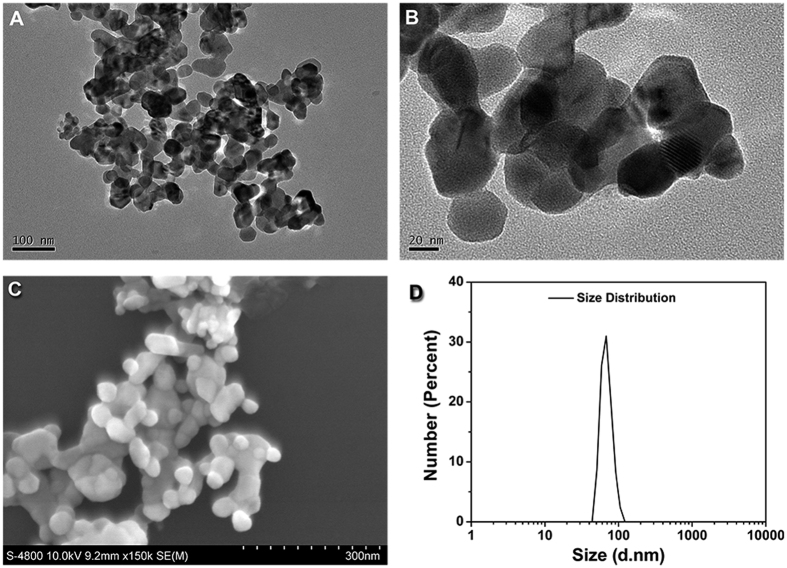
The physicochemical characterization of ZnONPs and ZnOMPs. (**A**) TEM images of ZnONPs. (**B**) SEM image of ZnONPs. (**C**) Size distribution of ZnONPs in Milli-Q water containing 1% sodium carboxymethylcellulose by DLS measurement. (**D**) Representative SEM image of ZnOMPs.

Representative TEM and SEM images show that the diameter of ZnONPs is 29.7 ± 4.0 nm (Fig. 1A and B). The ZnONPs were readily dispersed in 1% CMC solution, with a zeta potential of −59.4 ± 3.8 mV and a narrow size distribution of 69.4 ± 13.0 nm (Fig. 1C). The SEM images show the diameter of ZnOMPs is 701.3 ± 418.7 nm (Fig. 1D).

The releasing rates of Zn^2+^ from the particles in the suspension solution were evaluated. The data show that Zn^2+^ released from ZnONPs was very quick and was in a dose-dependent manner. Within 0.5~1 h post-dispersion, approximately 9.5, 13.8, and 23.2~26.5 μg/mL Zn^2+^ were detected in the supernatants of 1, 10 and 100 ZnONP suspensions, respectively; for 100 mg/mL ZnOMP suspension, 9.7 μg/mL Zn^2+^ were found in the supernatant, indicating that Zn^2+^ could quickly release from the surface of the particles and ZnONPs had much higher Zn^2+^ shedding rates than ZnOMPs (Supplementary Figure S1).

### Safety observation of ZnONPs on the mouse intestinal tract

The disease activity index (DAI) was used to estimate some early histopathological changes in colon of mice after ZnONP treatment (Supplementary Figure S2A). As the results, no significant increase in DAI was observed at 0.5, 5 and 50 mg/kg doses of ZnONP treatment (Figures S2B and C). Meanwhile, no obvious histological lesion and colon length changes in ZnONP-treated mice were observed (Supplementary Figure S2D and E). The results indicate that ZnONP treatment did not induce any obvious colitis-like clinic changes in mice at the tested doses.

### Effects of ZnONPs on DSS-induced colitis

**Figure 2:**
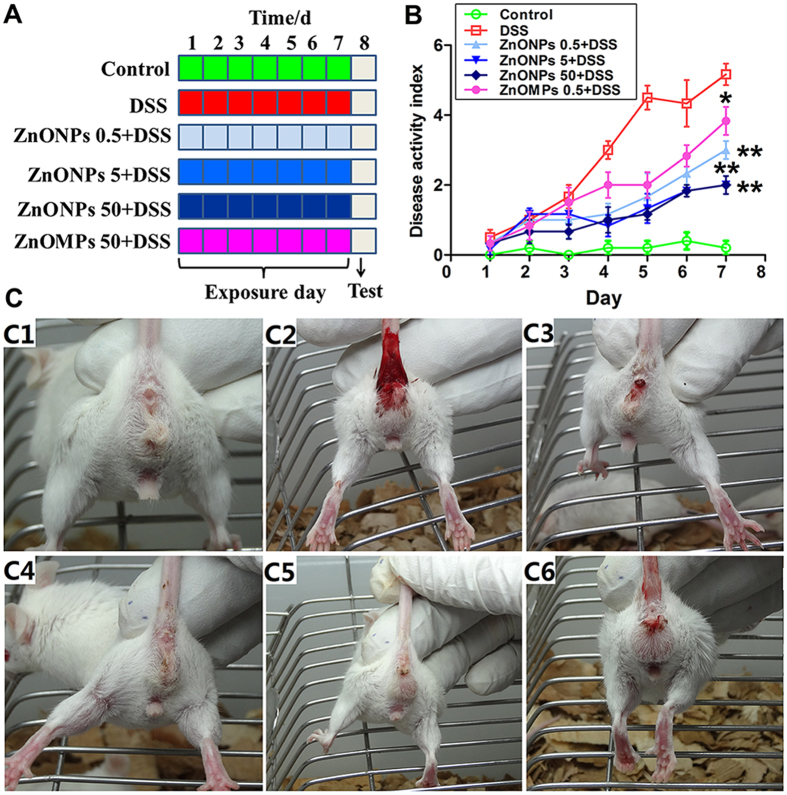
Therapeutic effect of ZnONPs on DSS-induced colitis in mice. (**A**) Experimental design of ZnONP treatment for DSS-injured mice. (**B**) Changes in DAI. Asterisks * and ** denote *p* < 0.05 and *p* < 0.01 compared with DSS group (n = 5), respectively. (**C**) Representative photographs of viable blood observation in mice. C1: Control; C2: DSS; C3: ZnONPs 0.5 + DSS; C4: ZnONPs 5 + DSS; C5: ZnONPs 50 + DSS; C6: ZnOMPs 50 + DSS groups.

**Figure 3:**
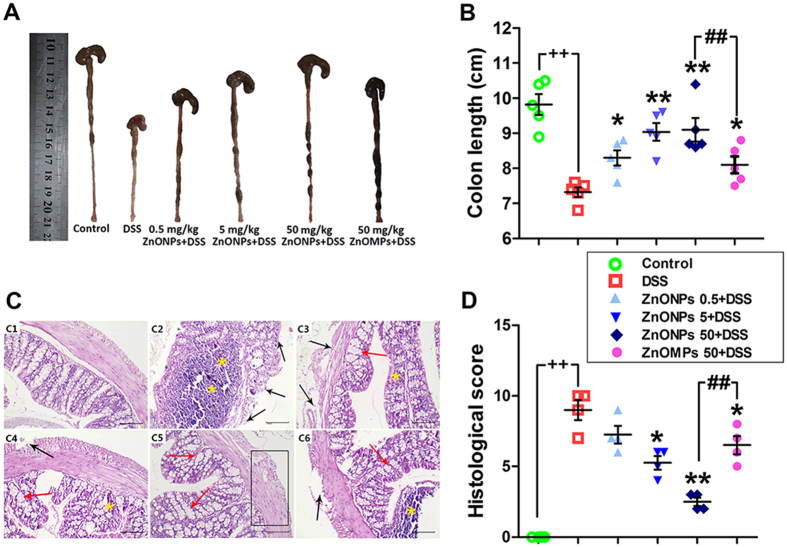
The effects of ZnONP/ZnOMP treatments on the colon length and histological changes of DSS mice. Representative images (**A**) and statistical analysis (**B**) of colon length. Representative H&E images (**C**) and histological scores (**D**) of the colon tissue in mice. Plus ^++^ denotes *p* < 0.01 compared with control group (n = 5); asterisks * and ** denote *p* < 0.05 and *p* < 0.01 compared with DSS group (n = 5), respectively; crosshatch ^#^ and ^##^ denote *p* < 0.05 and *p* < 0.01 compared with 50 mg/kg ZnOMPs + DSS group (n = 5), respectively. (**C**) Representative H&E-stained sections of colons, epithelial ulceration (black arrow), severe edema/inflammation (yellow asterisk), retention/regeneration of crypts (red arrow), and evidence of repair of the epithelium (box); scale bars, 100 μm.

To assess the efficiency of ZnONPs on colitis in mice, the DSS mice were orally administrated with ZnONPs for 7 days (Fig. 2A). The DSS-injured mice showed a significant increase in DAI, with visible blood in stool and marked colon shortening (Figs 2B,C and 3A,B). The obvious histological changes of epithelial ulceration, severe edema/inflammation and loss of crypts were found (Fig. 3C and D). As shown in Figs 2 and 3, ZnONP treatment can attenuate the DSS-induced colitis in mice with dose-dependent manner, including reduction DAI and histological lesion scores, and increase in colon length (*p* < 0.05, *p* < 0.01), indicating the potentially supportive therapy of ZnONPs on colitis.

**Figure 4:**
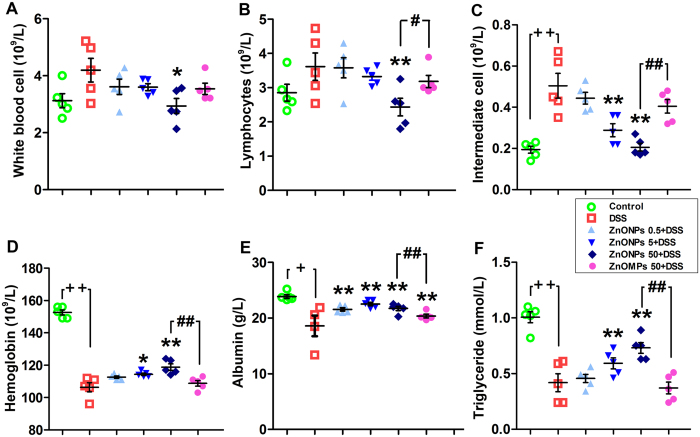
Blood hematologic and biochemical results of treated mice. After 7 days of treatment, blood hematologic (**A**–**C**) and biochemical (**D**–**F**) analysis were performed. Plus ^++^ denotes *p* < 0.01 compared with control group; asterisks * and ** denote *p* < 0.05 and *p* < 0.01 compared with DSS group, respectively; crosshatch ^#^ and ^##^ denote *p* < 0.05 and *p* < 0.01 compared with 50 mg/kg ZnOMPs + DSS group, respectively. n = 5.

The changes of blood hematological and serum biochemical values were monitored to evaluate the potential effects of ZnONPs on DSS-induced colitis in mice (Supplementary method). The high white blood cell, lymphocytes and intermediate cell counts and low hemoglobin, albumin and triglyceride contents showed that DSS treatment could affect the immune system and cause severe inflammatory responses in the mouse colon (Fig. 4). Interestingly, administration of 50 mg/kg ZnONPs significantly induced reduction in the number of white blood cell, lymphocytes and intermediate cell counts and also induced the elevation of hemoglobin, albumin and triglyceride levels with a dose-dependent manner (Fig. 4A–F, *p* < 0.05, *p* < 0.01). Comparatively, the similar treatment by ZnOMPs (50 mg/kg) showed lower efficacious than by ZnONPs (Figs 2, 3 and 4).

In the meantime, the efficacy of Zn^2+^ (75 μg/kg bw, which based on the amount of Zn^2+^ released from 2 mg/mL ZnONP suspension solution) against DSS-induced colitis was evaluated. The data show that the Zn^2+^ treatment helped reduce the DAI scores and alleviate the histological damage, but its efficiency was not as significant as ZnONP treatment (Supplementary Figure S3). The results suggest that the released Zn^2+^ may contribute to the attenuation of colonic damage by ZnONP treatment.

### Effects of combination therapy with ZnONPs and mesalazine on DSS-induced colitis

**Figure 5:**
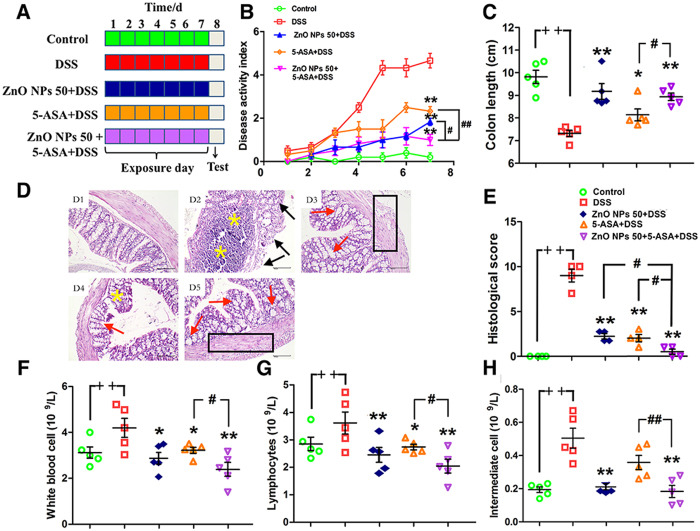
Effects of combination therapy of ZnONPs with 5-ASA on DSS-induced colitis in mice. (**A**) Experimental design. Changes in DAI (**B**) and colon length (**C**). Representative H&E images (**D**) and histological scores (**E**) of the colon tissues in mice. Mean and standard deviation of white blood cell count (**F**), lymphocytes count (**G**) and intermediate cell count (H) of mice by different treatments. Plus ^++^ denotes *p* < 0.01 compared with control group; asterisks * and ** denote *p* < 0.05 and *p* < 0.01 compared with DSS group, respectively; crosshatch ^#^ and ^##^ denote *p* < 0.05 and *p* < 0.01 compared with ZnONPs 50 + 5-ASA + DSS group, respectively. n = 5. (**D**) Representative H&E-stained sections of colons, D1: Control; D2: DSS; D3: ZnONPs 50 + DSS; D4: 5-ASA + DSS; D5: ZnONPs 50 + 5-ASA + DSS groups; epithelial ulceration (black arrow), severe edema/inflammation (yellow asterisk), retention/regeneration of crypts (red arrow), and evidence of repair of the epithelium (box); scale bars, 100 μm.

5-ASA is a bowel-specific antiulcer and is considered as an anti-inflammatory drug for clinical treatment of IBD^26^. The present results show that after 5-ASA therapy, the DAI and histological lesion scores had reduced and colon length increased in DSS-induced colitic mice (Fig. 5). Comparatively, the supportive therapy of 50 mg/kg ZnONPs (ZnONPs 50) with 5-ASA was more efficacious than 5-ASA or ZnONPs 50, respectively, with more significant reduction in DAI and increasing in colon length in DSS-induced colitis mice (Fig. 5B and C; *p* < 0.05, *p* < 0.01). The histopathological analysis of the colon tissues shows a more effective supportive effect of ZnONPs 50 for 5-ASA treatment compared with only 5-ASA or ZnONPs 50 treatment, the evidence includes less inflammatory infiltration, more regeneration of crypts and complete epithelial in the colon of DSS mice (Fig. 5D and E). Correspondingly, the levels of white blood cell, lymphocytes and intermediate cell counts significantly decreased by the combination therapy (Fig. 5F–H, *p* < 0.05, *p* < 0.01). The results demonstrate that ZnONPs can enhance the antiulcer activity of 5-ASA on DSS-induced colitis in mice.

### Treatment with ZnONPs reduces oxidative stress in the colon of DSS-induced colitis in mice

**Figure 6:**
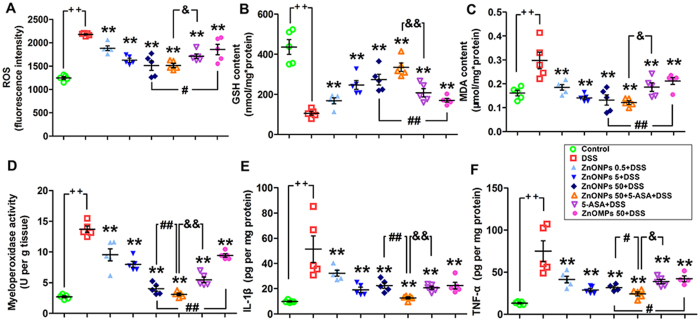
The anti-oxidative and anti-inflammatory activity of ZnONPs in colon of colitic mice. Levels of ROS (**A**), GSH (**B**), MDA (**C**), MPO activity (**D**), IL-1β (**E**) and TNF-α (**F**) in colon of mice. Plus ^++^ denotes *p* < 0.01 compared with control group; asterisks * and ** denote *p* < 0.05 and *p* < 0.01 compared with DSS group, respectively; crosshatch ^#^ and ^##^ denote *p* < 0.05 and *p* < 0.01 compared with 50 mg/kg ZnONPs + DSS group, respectively; ampersand ^&^ and ^&&^ denote *p* < 0.05 and *p* < 0.01, compared with 5-ASA + DSS group, respectively. n = 5.

The levels of ROS and MDA significantly increased, and GSH significantly decreased in the colon of DSS-mice as compared to the control group, indicating that DSS treatment induced significant oxidative damage in the colon of mice (Fig. 6A–C, *p* < 0.01). The ZnONP treatments (0.5, 5, 50 mg/kg) significantly reduced the levels of ROS and MDA, and increased GSH in colon of DSS mice in a dose-dependent manner (Fig. 6A–C, *p* < 0.05). The results demonstrate that ZnONPs can exert antioxidative effects on DSS-induced colitis in mice. It is worth noting that the combination therapy of ZnONPs 50 + 5-ASA shows the highest antioxidative efficacy among the single 5-ASA, ZnONPs 50 and ZnOMPs 50 treatments (Fig. 6A–C).

In the study, the myeloperoxidase (MPO) activity in DSS-induced colitic mice elevated about four times as compared with the control mice (Fig. 6D), which is consistent with the previous studies^27^. The oral administration of single ZnONPs and ZnONPs + 5-ASA combination significantly reduced MPO activity in the DSS-mice (Fig. 6D, *p* < 0.01). To determine the effect of ZnONPs on pro-inflammatory cytokine expression, colonic interleukin (IL)-1β, and tumor necrosis factor (TNF-α), which are well-known markers of inflammation and play an important role in UC^27^, were determined. The data show that ZnONP treatment significantly decreased the expression of IL-1β and TNF-α in DSS-treated colon (Fig. 6E and F, *p* < 0.01). Moreover, ZnONPs can enhance the anti-inflammatory effects of 5-ASA against DSS-induced colitis in mice (Fig. 6D and E, *p* < 0.01; Fig. 6F, *p* < 0.05). Comparatively, the anti-inflammatory effect of ZnONPs is better than that of ZnOMPs (Fig. 6D and E, *p* < 0.01; Fig. 6F, *p* < 0.05).

These results demonstrate the potential anti-oxidant and anti-inflammatory effects of ZnONPs against DSS-induced colitis in mice, especially of their combination with 5-ASA.

### Modulation of Nrf2 and NQO-1 expression by ZnONPs against DSS-induced colitis in mice

**Figure 7:**
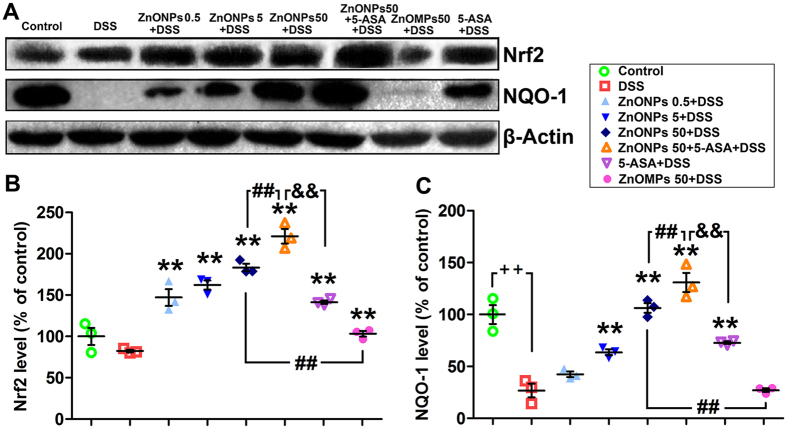
Expression of Nrf2 and NQO-1 in the colon of colitic mice. (**A**) Western blotting analysis of the expression change of Nrf2 and NQO-1 proteins in the colon of mice, β-actin was used as a housekeeping control; Full-length gels are presented in Supplementary Figure S9. (**B**,**C**) The statistical analysis showed that ZnONP treatment led to a significant increase in the expression of Nrf2 and NQO-1 in the colon of DSS-induced colitis mice. All protein expression levels were normalized to housekeeping control. Plus ^++^ denotes *p* < 0.01 compared with control group; asterisks * and ** denote *p* < 0.05 and *p* < 0.01 compared with DSS group, respectively; crosshatch ^#^ and ^##^ denote *p* < 0.05 and *p* < 0.01 compared with 50 mg/kg ZnONPs + DSS group, respectively; ampersand ^&^ and ^&&^ denote *p* < 0.05 and *p* < 0.01, compared with 5-ASA + DSS group, respectively. n = 5.

Nrf2 is an important cytoprotective transcription factor and can induce the expression of antioxidant enzymes, such as NQO-1, which can attenuate oxidative and inflammatory damage^28^,^29^. DSS treatment induced reduction of Nrf2 and NQO-1 expressions compared with the control group in colon tissues (Fig. 7). Interestingly, ZnONP treatment led to significantly dose-dependent up-regulation of Nrf2 and NQO-1 in colitic colon (Fig. 7B and C, *p* < 0.05). Comparatively, the combination treatment of ZnONPs 50 + 5-ASA induced the most obvious results. In contrast, the level of Nrf2 and NQO-1 expression in colon tissues in ZnOMP-treated mice was lower than that in ZnONP-treated ones (Fig. 7B and C, *p* < 0.01). These results indicated that ZnONPs protected against DSS-induced colitis by activating the Nrf2 signaling pathway.

### Effect of ZnONPs on colonic microflora in DSS-induced colitis mice

**Figure 8:**
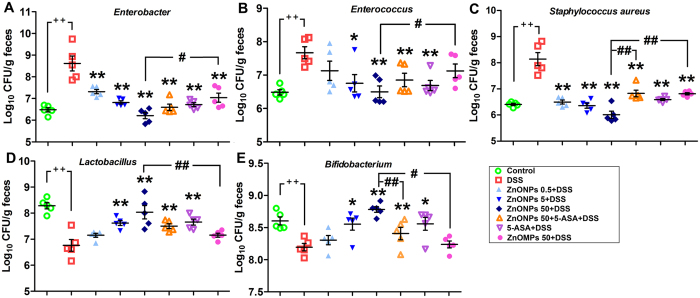
Colonic microflora analysis in the treated mice (n = 5). Statistical analysis of the number of *Enterobacter* (**A**), *Enterococcus* (**B**), *Staphylococcus aureus* (**C**), *Lactobacillus* (**D**) and *Bifidobacterium* (**E**) in the colon of mice. Plus ^++^ denotes *p* < 0.01 compared with control group; asterisks * and ** denote *p* < 0.05 and *p* < 0.01 compared with DSS group, respectively; crosshatch ^#^ and ^##^ denote *p* < 0.05 and *p* < 0.01 compared with 50 mg/kg ZnONPs + DSS group, respectively.

Several evidences suggest that the gut microbiota play a vital role in the pathogenesis of IBD^30^. The changes of gut bacteria may contribute to the development of DSS-induced colitis^30^,^31^. To investigate the alterations of gut bacteria composition in colitis mouse model, the relative abundance of five common gut bacterium species in mouse colon, including *Enterobacterium, Enterococcus, Staphylococcus aureus, Lactobacillus* and *Bifidobacterium* were analyzed (Supplementary Figures S3–S7). The data show that the number of *Enterobacterium, Enterococcus* and *Staphylococcus aureus* significantly increased, meanwhile, the number of probiotics of *Lactobacillus* and *Bifidobacterium* significantly decreased in colon of DSS-injured mice compared with the control group (Fig. 8, *p* < 0.01). The 5-SAS therapy effectively decreased the number of *Enterobacterium, Enterococcus* and *S. aureus*. Interestingly, the treatments of ZnONPs alone decreased the number of *Enterobacterium, Enterococcus* and *S. aureus*, meanwhile, increased the probiotics *Lactobacillus* and *Bifidobacterium* in a dose-dependent manner. The combination therapy of 50 mg/kg ZnONPs + 5-ASA induced the similar antibacterial effects. In contrast, ZnONPs show more effective antibacterial activity than the similar dose of ZnOMPs (Fig. 8, *p* < 0.01).

## Discussion

IBD (includes UC and CD) is a chronic intestinal disorder of the gastrointestinal tract that affects millions of patients worldwide^1^. It is reported that after 30 years of living with the diseases, UC and CD patients pose a risk of developing colitis-associated colon cancer, the third most common malignancy and one of the major causes of cancer related death in humans^6^. Up to now, the precise of the initiation and/or propagation of the inflammation for IBD is still unknown and is difficult to remove. Many efforts have been made to find effective therapeutic treatments for the disease. Mesalazine (5-ASA) is the first-line therapy for IBD, especially for maintenance of remission^32^. The pharmacodynamic studies show that 5-ASA inhibits inflammatory cell functions, plasma cell antibody production and TNF activity, decreases IL-1 production, and acts as a free oxygen radical scavenger^33^. More importantly, a large amount of molecular data support the notion that 5-ASA acts as chemopreventive compounds to interfere with colorectal cancer development, though the exact mechanism remains unknown^26^. However, although 5-ASA preparations are generally well tolerated by most patients, adverse reactions after high dosage and long term intake have been described, including nausea, vomiting, worsening colitis, and rarely nephrotoxicity, hepatotoxicity, pericarditis, pancreatitis, etc^33^. Therefore, a supportive therapy agent that can increase the curative effect is urgently required.

The DSS-induced colitis in mice is one of the commonly used murine models of IBD, which is very similar to human ulcerative colitis^30^,^34^. Our study shows that therapy by 5-ASA, as expected, obviously attenuated the severity of colitis in mice, including decrease DAI, increase colon length and alleviate the damage of colonic mucosal, furthermore, showed anti-inflammatory effects that suppressed proinflammatory mediators of MPO, IL-1β and TNF-α. The interesting result is that the ZnONP treatments showed markedly dose-dependent effects on the remission of the disease. In this study, we confirm that ZnONPs can antagonize oxidative stress and inflammatory responses, and importantly, restore the balance of intestinal flora in DSS-induced colitis mice. Activating Nrf2 pathway is probable to be the underlying molecular mechanism of ZnONP treatment in the cellular antioxidant defense system. The combination therapy ZnONPs with 5-ASA on DSS-induced colitis can enhance the antiulcer activity of 5-ASA, demonstrating a potentially supportive therapy of ZnONPs for the treatment of IBD.

Oxidative stress is hypothesized as a potential etiological and/or triggering factor for IBD, though the molecular pathways of colon cell damage are not completely understood^7^. Clinical data show that ROS increases in IBD patients, causing oxidative cellular damage and promoting carcinogenesis^7^. In the study, the DSS injury induced significant elevation of ROS production; decreased GSH and increased MDA levels in colon tissues, indicating the colitis in mice is correlated with oxidative stress. Meanwhile, significant high concentrations of proinflammatory cytokines IL-1β and TNF-α were generated, which are implicated in the progression of UC^35^. ZnONPs have gained a great advantage to apply in medical and nutritional applications due to their unique properties and biological safety^23^. Several actions of ZnONPs have been reported to the beneficial effects on human health, such as anticancer, antimicrobial and antioxidant activities^23^,^36^. In the study, the ZnONP treatments show significant dose-dependent efficacy for reduction of oxidative stress in colon of colitic mice, and meanwhile, suppress proinflammatory cytokines (IL-1β and TNF-α) and MPO, indicating ZnONPs possess anti-oxidant and anti-inflammatory activities. The 5-ASA treatments, as known, significantly reduced the oxidative stress levels, which is consistent with the previous reports^26^,^32^. The important finding is that the combination therapy of ZnONPs 50 + 5-ASA is more effective than monotherapy of ZnONPs and 5-ASA. Therefore, it is interesting to explore whether ZnONPs and 5-ASA involve in the similar antioxidant pathway in colitic mouse.

Nrf2 is a basic leucine zipper redox sensitive transcriptional factor that plays an important role in antioxidant response element-mediated induction of diverse antioxidant enzymes^3^,^11^,^29^. Khor *et al*. found that mice lacking Nrf2 were more susceptible to DSS-induced colitis^3^. The Nrf2^−/−^ mice was found to be associated with more intense of proinflammatory cytokines, such as IL-1β and TNF-α, and more severe colonic colitis, including loss of colonic crypt, occurrence with massive infiltration by inflammatory cells than WT mice^29^. The similarly findings were found in the DSS-mice the colonic colitis increased in IL-1β and TNF-α, and reduced the expression of protein Nrf2 and phase II protein NQO-1, indicating DSS treatment decreased Nrf2 activation. Zinc is known as a major component of the cell antioxidant network that maintains cell redox homeostasis through various mechanisms including the regulation of antioxidant protective responses of Nrf2^18^. The ZnONP treatments led to a dose-dependent increase in the expression of Nrf2 and anti-oxidant enzyme NQO-1 in the colon of colitic mice, demonstrating the activation of Nrf2 was involved in the response of ZnONPs acting as antioxidants in the colitic mice. The noteworthy result is that ZnONPs have more effective antioxidant activity than ZnOMPs when treated to the colitic mice. These results are in consistent with the previous findings that ZnONPs display extremely high scavenging capacity of free radicals due to their large surface area and high catalytic activity of the nanoparticles^37^. The antioxidative activity of 5-ASA show correlation with the responses of Nrf2 activation, but the levels of Nrf2 and NQO-1 expression are lower than ZnONPs-50 and higher than ZnOMPs-50 treatments. However, the combination therapy of 5-ASA with ZnONPs induced relatively the highest increased expression of Nrf2 and NQO-1, demonstrating that the combination therapy of 5-ASA with ZnONPs attenuates oxidative stress in colitis mice by modulating Nrf2 signaling pathways.

Recent studies have demonstrated that the resident intestinal bacteria play a role in initiating and maintaining IBD^38^. The disruption of the gut microbiota may drive the abnormal inflammatory response in the disease^31^,^38^. Antibiotics or antimicrobial peptide has shown to ameliorate or prevent inflammation in patients and in murine models of IBD, demonstrating the major impact of gut microbiota on disease pathogenesis^30^. For instant, a marked reduction in antimicrobial activity against *B. vulgatus, E. coli*, and *E. faecalis* was observed in extracts from Crohn patients^39^. The administration of a mixture of probiotics: *Lactobacillus, Bifidobacterium* and *Streptococcussalivarious* strains, has shown to have beneficial effects on colitis mouse models and IBD patients^40^. Our study shows that ZnONPs possess effects of supportive therapy for DSS-induced colitis in mice. However, the influence of ZnONPs on the colonic microflora remains unknown. The analysis of colonic microflora indicates that the DSS injury led to the changes in the intestinal microbiota of mice, *i.e.*, the relative abundance of *Enterobacter, Enterococcus* and *S. aureus* increased and the probiotics, such as *Lactobacillus* and *Bifidobacterium* reduced. Interestingly, the ZnONP treatments alone obviously alter in the community of the gut bacteria in colon of the colitis mice, especially, in a dose-dependent manner, significantly increased the number of *Lactobacillus* and *Bifidobacterium*, of which are known as probiotics that have antagonistic effects against pathogenic bacteria in biological system, and in the meantime, decreased the numbers of *Enterobacter, Enterococcus* and *S. aureus*. In contrast, as an anti-inflammatory drug, 5-ASA treatment significantly decreased all the tested gut bacteria, including both the pathogens and probiotics, which is consistent with the previous reports^26^. Notably, the alteration of the gut microbacteria by the ZnONPs, 5-ASA and ZnONPs 50 + 5-ASA treatments accompany with the remission of the severity of colitis in mice. It is known that the invading bacteria, such as *E. coli*, contribute to inflammation by adhering to and invading epithelial cell lines and human ileal mucosa^38^,^41^; persist and replicate in macrophages, and secrete large quantities of TNF^38^. Our results indicate that the anti-inflammatory effect of ZnONPs associate with the reduction of translation and infection by commensal bacteria in the colonic mucosa. Since ZnONPs could also enhance the number of probiotics like *Lactobacillus* and *Bifidobacterium* in colon of colitis mice, ZnONP treatment can be applied in nutritional or supportive therapy in the treatment of IBD. The results suggest that the novel therapeutic strategy of ZnONPs conjunction with traditional drug (*e.g.*, 5-ASA) could enhance the effectiveness of IBD therapy.

Additionally, the research demonstrates that ZnONPs are more efficacious than ZnOMPs in the treatment of colitis in mice. The results may due to several reasons: 1) ZnONPs have stronger antioxidant and anti-inflammatory activity than ZnOMPs; 2) restoration on the alteration of the abnormal intestinal microbiota by ZnONPs is more effective than by ZnOMPs; 3) ZnONPs are easier diffusion in the mucosa compared with larger-sized particles^42^, and more zinc ions may release from the nano-surface than micro-surface^43^, thus shows more obvious efficacy than that by ZnOMPs. The results indicate that ZnONPs is a promising zinc species with potential application in attenuation of IBD.

In summary, our study demonstrates that ZnONPs could attenuate DSS-induced colitis in mice, including reduction in DAI and histological lesion score, increase in colon length, which has the better therapeutic effects than that of ZnOMP treatment. The mechanism of the above effects may partially be due to the ability of ZnONPs to improve the colonic anti-oxidant and anti-inflammatory potential *via* suppressing ROS and MDA production; induction the antioxidant enzyme of GSH; meanwhile, suppression of proinflammatory cytokines (IL-1β and TNF-α) and MPO in the colitic mice. In addition, ZnONP treatment could induce upstream regulation of Nrf2 and NQO-1, which are the proteins representative antioxidant and cytoprotective factors involved in colitis and cancer chemoprevention^12^. Importantly, ZnONP treatment is first found beneficial to restore the unhealthy change of the intestinal microbiota in colitic mice, which has been demonstrated to be a vital role in initiating and maintaining IBD^38^. The combination therapy of ZnONPs with 5-ASA on DSS-induced colitis shows that ZnONPs can enhance the antiulcer activity of 5-ASA. Our results collectively suggest that ZnONPs acts as a supportive therapy for the treatment of IBD.

## Methods

### Preparation of ZnONP and ZnOMP suspension solution

ZnONPs and ZnOMPs were purchased from Hangzhou Wanjing New Material Co. Ltd (Hangzhou, China). Before animal experiments, ZnONPs were dispersed in 1% sodium carboxymethylcellulose (CMC) solution and the suspensions were sonicated at 12–15 °C for 0.5 h. Finally, the 10 mg/mL well-dispersed ZnONP and ZnOMP suspension solutions were obtained. For animal treatments, the suspension solution was further diluted with deionized water and sonicated for 15 min.

### Animal

Male BABL/c mice (7-week-old, approximately 20 g) were purchased from Vital River Laboratory Animal Technology Co. Ltd. (Beijing, China). The mice were housed two per cage at 24–26 °C with 60 ± 2% humidity and a 12-h light-dark cycle, having *ad libitum* access to water and food. All animal experiments were performed according to the guidelines for ethical conduct in the care and use of animals in research by Chinese Society of Toxicology, and were approved by the Office of Scientific Research Management of Institute (March 1, 2012 CCNU-IACUC-2012-011).

### DSS-induced colitis in mice

The experimental acute colitis was induced in mice by feeding 3% (w/v) DSS (M.W. = 36,000–50,000 Da; MP Biomedicals) in their drinking water for 7 days.

### Protective role of ZnONPs against colitis in mice

A total of 30 male mice were randomly divided into 6 groups (n = 5 per group) and treated as follows: (a) Control group (Control); (b) 3% DSS-induced colitis group (DSS); (c) 0.5 mg/kg bw ZnONPs + DSS group (ZnONPs 0.5 + DSS); (d) 5 mg/kg bw ZnONPs + DSS group (ZnONPs 5 + DSS); (e) 50 mg/kg bw ZnONPs + DSS group (ZnONPs 50 + DSS); and (f) 50 mg/kg bw ZnOMPs + DSS group (ZnOMPs 50 + DSS). The particle suspension was orally administrated to DSS mice once a day for 7 days.

As a comparison, the effects of Zn^2+^ that released from ZnONPs on DSS mice were estimated (Supplementary Data).

### Therapeutic effect of combination ZnONPs with mesalazine against colitis in mice

The mice were randomly divided into 5 groups (n = 5): (a) Control group (Control); (b) 3% DSS-induced colitis group (DSS); (c) 50 mg/kg bw ZnONPs + DSS group (ZnONPs 50 + DSS); (d) 5-ASA + DSS group (5-ASA + DSS) and (e) 50 mg/kg ZnONPs + 5-ASA + DSS group (ZnONPs 50 + 5-ASA + DSS). The drug 5-ASA (200 mg/kg/day) and ZnONP suspensions were orally administered daily to the mice for 7 days.

### DAI and colon length measurements

During 7 days of treatment, the changes in body weight, visible stool consistency and fecal bleeding were recorded daily. Disease activity index (DAI) is the summation of the stool consistency index (0–3); fecal bleeding index (0–3); and weight loss index (0–4)^44^. After 7 days of treatment, mice were anesthetized with diethyl ether and the entire colon (from cecum to rectum) was collected. Colon was gently washed with PBS (pH = 7.4) and the length was measured and recorded.

### Colonic bacteria analysis

Freshly sterile colonic samples were collected and homogenized in ice-cold PBS (pH 7.4) in a glass homogenizer. 10-fold serial dilutions (10^−1^–10^−7^) were performed in medium. The intestinal bacteria: *Enterobacter, Enterococcus* and *Staphylococcus aureus* were cultured on Eosin-Methylene Blue agar, Enterococcus Agar and Mannitol Salt Agar (Qingdao Nissui Biological Technology Co. Ltd., Qingdao, China), respectively; *Lactobacillus* and *Bifidobacterium* were cultured on Lactobacillus Selective Agar and TPY Agar (Qingdao Nissui Biological Technology Co. Ltd., Qingdao, China) in an anaerobic jar containing an AnaeroGen^TM^ bag (Oxoid), respectively. The colonies were incubated at 37 °C for 24–48 h and the counts were expressed as log CFU/g.

### Statistical analysis

Data are presented as mean ± SD. Statistical graphs were generated using GraphPad Prism 5.0. One-way ANOVA combined with Fisher’s protected *t*-test were used to determine the statistical differences between groups. *p* < 0.05 and *p* < 0.01 were considered significant.

## Additional Information

**How to cite this article**: Li, J. *et al*. ZnO nanoparticles act as supportive therapy in DSS-induced ulcerative colitis in mice by maintaining gut homeostasis and activating Nrf2 signaling. *Sci. Rep.* **7**, 43126; doi: 10.1038/srep43126 (2017).

**Publisher's note:** Springer Nature remains neutral with regard to jurisdictional claims in published maps and institutional affiliations.

## Supplementary Material

Supplementary Data
