# Supplementary material for: ZnO nanoparticles act as supportive therapy in DSS-induced ulcerative colitis in mice by maintaining gut homeostasis and activating Nrf2 signaling
Source: Sci Rep. 2017 Feb 24;7:43126. doi: 10.1038/srep43126 (PMC5324050; doi:10.1038/srep43126)
Supplement: Supplementary Data [file srep43126-s1.pdf]

Supplementary Data

ZnO nanoparticles act as supportive therapy in DSS-induced ulcerative colitis in mice by maintaining gut homeostasis and activating Nrf2 signaling

Jinquan Li[1, 2+], Hanqing Chen[1+], Bing Wang[1], Chengxu Cai[1, 3], Xu Yang[2*], Zhifang Chai[1], Weiyue Feng[1*]

[1] CAS Key Laboratory for Biomedical Effects of Nanomaterials and Nanosafety, Institute of High Energy Physics, Chinese Academy of Sciences (CAS), Beijing 100049, China.

[2] Section of Environmental Biomedicine, Hubei Key Laboratory of Genetic Regulation and Integrative Biology, College of Life Sciences, Central China Normal University, Wuhan, 430079, China.

[3] National Center for Nanoscience and Technology, Beijing, 100190, China.

[+] These authors contributed equally to this work.

*Corresponding author: fengwy@ihep.ac.cn (Weiyue Feng);

yanxgu@mail.ccnu.edu.cn (Xu Yang).

Methods

Physicochemical characterization of ZnONPs and ZnOMPs

The size and shape of ZnONPs were characterized by transmission electron microscopy (TEM, JEOL JEM-2010, Japan), scanning electron microscopy (SEM, Hitachi S-4800, Japan). The size distribution and zeta potential of ZnONPs were determined by dynamic light scattering (DLS) using Malvern Zetasizer (Zetasizer Nano ZS, UK).

Measurement of Zn^{2+} release from ZnONP and ZnOMP suspension solution

The Zn^{2+} release from ZnONP (1, 10, 100 mg/mL) and ZnOMP (100 mg/mL) suspension solution was evaluated at 0.5, 1, 2, 4, 8 and 24 h post-dispersion using inductively coupled plasma-mass spectrometry (ICP-MS, Elemental X7, Thermo Electron Co., Waltham, USA). Briefly, after ZnONP dispersion in 1% CMC solution, NP-free supernatants were collected by 15,000 rpm centrifugation for 30 min at 4°C. The concentrations of zinc in supernatants were determined by the method of ICP-MS.

Animal experiments

Animals

Male BABL/c mice (7-week-old, approximately 20 g) were purchased from Vital River Laboratory Animal Technology Co. Ltd. (Beijing, China). The mice were housed two per cage at 24-26°C with 60 ± 2% humidity and a 12-h light-dark cycle, having *ad libitum* access to water and food. All animal experiments were performed according to the guidelines for ethical conduct in the care and use of animals in research by Chinese Society of Toxicology, and were approved by the Office of Scientific Research Management of Institute of High Energy Physics, CAS.

Histological assessment of colonic damage

After washing, all sections of the distal colon was fixed in 10% formalin solution for 24 h at room temperature and cut into 1 cm pieces for H&E staining. The section was observed by using the DM4000B microscope (Leica Microsystems GmbH, Wetzlar, Germany) and the histological lesion score was used to evaluate evidence of colitis in animals.

A histological score reflecting infiltration of inflammatory cells and epithelial structure was graded on a scale from 0 to 4 as follows: 0 = no evidence of inflammation; 1 = low level of inflammatory cell infiltration, with infiltration seen in ≤ 10% of high-power fields (HPFs) and no signs of epithelial degeneration; 2 = moderate inflammation, with inflammatory cell infiltration seen in 10-25% of HPFs, and multiple foci and/or mild epithelial ulcerations; 3 = moderate to severe inflammation, with inflammatory cell infiltration seen in 25-50% of HPFs, and marked wall thickening and/or ulcerations in > 30% of the tissue; and 4 = severe inflammation, with inflammatory cell infiltration seen in ≥ 50% of HPFs, and ulcerations of more than 75% of the tissue section[1].

Blood biochemical assay

Blood samples (0.5-1 mL) were collected from the orbital venous plexus into centrifuge tubes or sodium citrate coated tubes. Blood hematological and serum biochemistry parameters were measured on automatic chemistry analyzer (Celltac, MEK-6358; Nihon Kohden Co, Tokyo, Japan).

Measurement of ROS, reduced glutathione (GSH) and malondialdehyde (MDA) concentrations in colon tissues

Supernatant of colon tissue homogenate was diluted 2-fold in PBS, and 100 μL

of diluted supernatant mixed with 100 μL 2’,7’-dichlorofluorescein diacetate (DCFH-DA, Beytime Biotechnology, China). The reaction mixture was placed in the 96-well microplate in dark at 37°C for 30 min and the DCFH fluorescence was determined by a fluorescence microplate reader with 485 and 520 nm for excitation and emission.

GSH and MDA concentrations in colon tissue homogenate were measured using the previously described procedures[2]. The protein concentration was determined using the Modified BCA Protein Assay Kit (Beytime Biotechnology, China).

Determination of myeloperoxidase (MPO) activity

Approximately 100 mg tissues from the colon region were snap-frozen in liquid nitrogen and homogenized in 1.0 ml potassium phosphate buffer containing 0.5% hexadecyltrimethyl ammonium bromide (HTAB). Tissue particulates were discarded by centrifugation (5,000 rpm, 2 min) and the supernatant was collected. MPO activity in supernatant was measured using o-dianisidine as substrate. The rate of change in absorbance was measured at 460 nm, and MPO activity was expressed as units per gram of tissue.

Measurement of IL-1β and TNF-α level

Tissues from the colon region were homogenized in a glass homogenizer on ice, using 10 mL/g of ice-cold PBS at pH 7.4 to produce a 10% tissue homogenate. Half of the homogenate was centrifuged at 10,000 rpm for 10 min at 4°C and the supernatant was collected and kept at -70°C. The tissue levels of IL-1β and TNF-α were analyzed by the commercial EILSA kits (eBioscience, San Diego, CA, USA).

Safety study of ZnONP treatment on intestinal tract of mice

In order to evaluate the biosafety of ZnONPs to intestinal tract of mice, twenty

mice were randomly divided into 4 groups (n = 5 per group): (a) Control group (Control); (b) 0.5 mg/kg body weight (bw) ZnONPs group (ZnONPs 0.5); (c) 5 mg/kg bw ZnONPs group (ZnONPs 5); and (d) 50 mg/kg bw ZnONPs group (ZnONPs 50). The ZnONPs were orally administrated to mice once a day for 7 days.

Estimate therapeutic effects of released Zn^{2+} against colitis in mice

To compare the therapeutic effect of ZnONPs versus the amount of Zn^{2+} that released from ZnONPs in suspension solution, a total of 24 mice were randomly divided into four groups (n = 6 per group): (1) Control group (Control); (2) 3% DSS-induced colitis group (DSS); (3) 75 μg/kg bw Zn^{2+} + DSS group (Zn^{2+} + DSS); (4) 50 mg/kg ZnONPs + DSS group (ZnONPs + DSS). The treated solutions were orally administrated to DSS mice once a day for 7 days.

Western blot analysis

Colons were washed with PBS for three times and lyzed with protease inhibitor containing RIPA buffer (50 mM Tris-HCl, pH 7.4, 150 mM NaCl, 1.0% Triton X-100, 1% sodium deoxycholate, 0.1% SDS) for 15 min at 4°C. The homogenate was mixed with 5 × loading buffer and denatured for 5 min at 100°C. The samples were separated on 10% SDS-PAGE gel, and then transferred to PVDF membranes. Membranes were probed with the primary antibodies of rabbit anti-Nrf2, NQO-1 and β-actin (GeneTex, Inc., USA).

References

1. Zwiers A, Fuss IJ, Seegers D, Konijn T, Garcia-Vallejo JJ, Samsom JN, et al. A Polymorphism in the Coding Region of Il12b Promotes IL-12p70 and IL-23 Heterodimer Formation. *J. Immunol.* 2011;**186**:3572-3580.
2. Li J, Li L, Chen H, Chang Q, Liu X, Wu Y, et al. Application of vitamin E to antagonize SWCNTs-induced exacerbation of allergic asthma. *Sci Rep* 2014;**4**:4275.

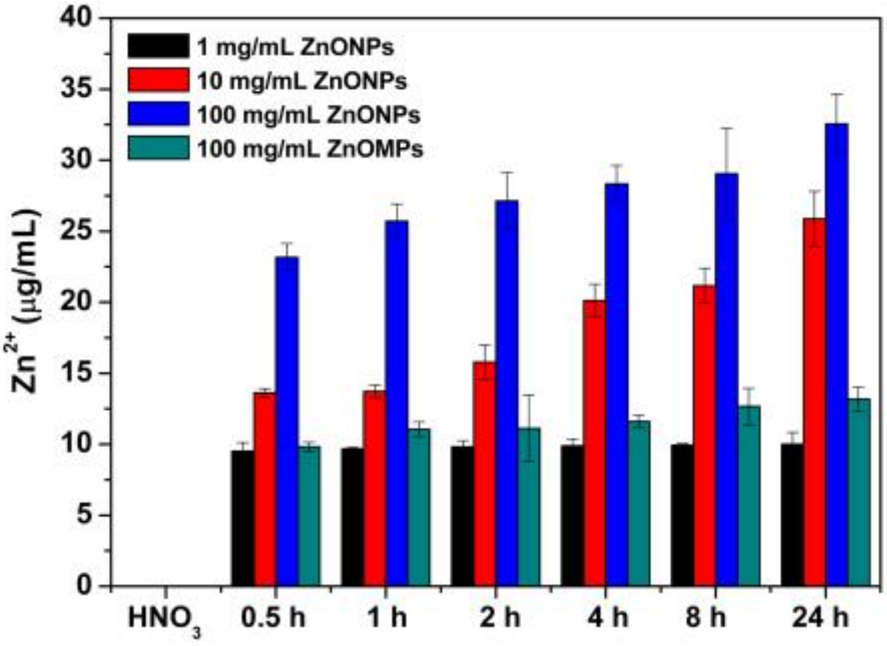

Figure S1. Time- and content-dependent Zn^{2+} releasing from ZnONP suspension solution (containing 1% sodium carboxymethylcellulose) by ICP-MS determination.

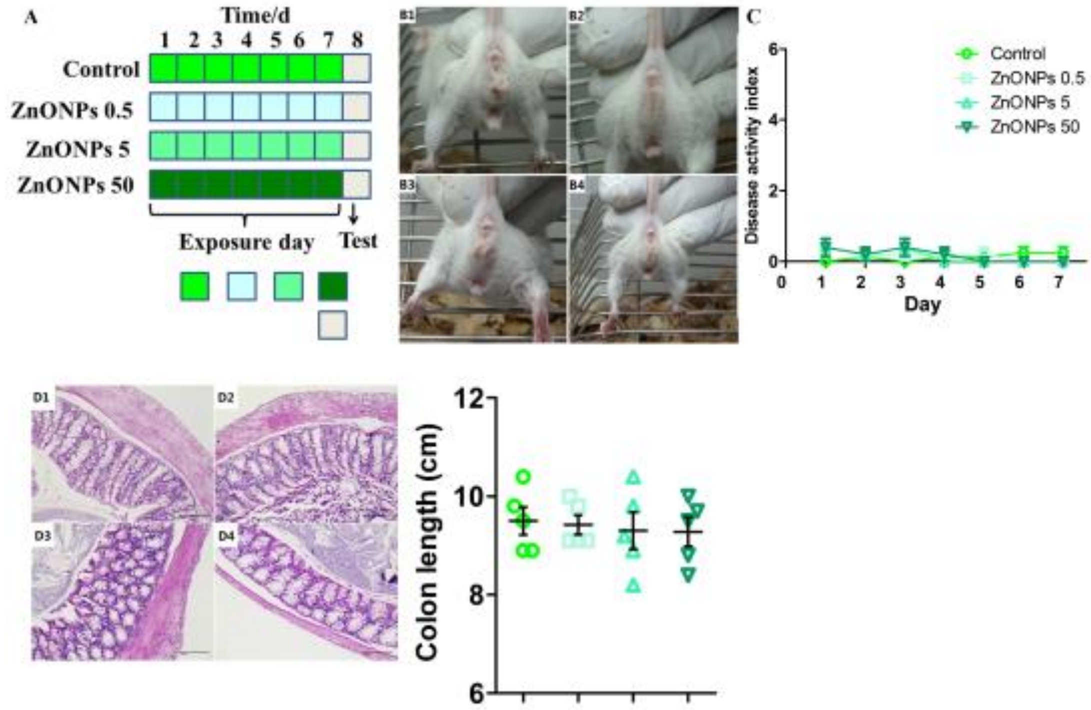

Figure S2. Effects of ZnONPs on intestinal tract of mice. (A) Experimental design. The animals were divided into four groups, 1% carboxymethyl sodium nitrate was orally administered daily for 7 days as control group; three different concentrations of ZnONPs (0.5, 5 and 50 mg/kg) were orally administered daily for 7 days (n = 5 mice per group). (B) Symptoms of the mice after 7-day ZnONP treatment. (C) Changes in DAI. Data are expressed as mean ± SE. (D) Representative photomicrographs of mice colon sections (hematoxylin & eosin staining, scale bars: 100 μm); D1–D4 represent different exposure groups: control; 0.5 mg/kg ZnONPs; 5 mg/kg ZnONPs and 50 mg/kg ZnONPs treated groups. (E) Changes in colon length (data are expressed as mean ± SE).

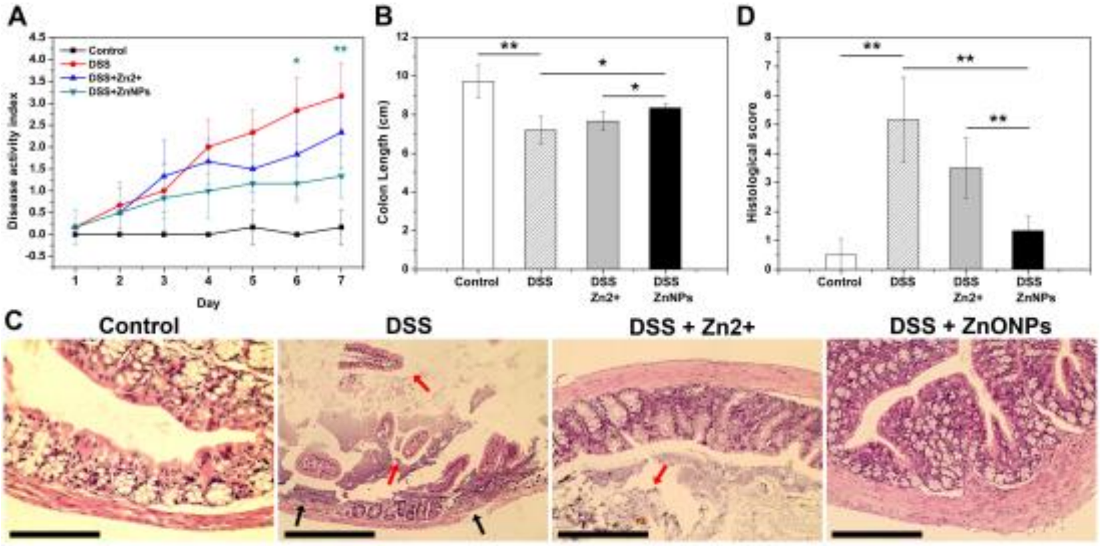

Fig. S3. Therapeutic effects of ZnONPs and released zinc ions on the colon length and histological changes of DSS mice. (A) Changes in DAI. Asterisks * and ** denote $p< 0.05$ and $p< 0.01$ compared with DSS group (n = 6), respectively. (B) Statistical analysis of colon length. Representative H&E images (C) and histological scores (D) of the colon tissue in mice (n = 6). Epithelial ulceration (black arrow), retention/regeneration of crypts (red arrow); scale bars, 100 μm.

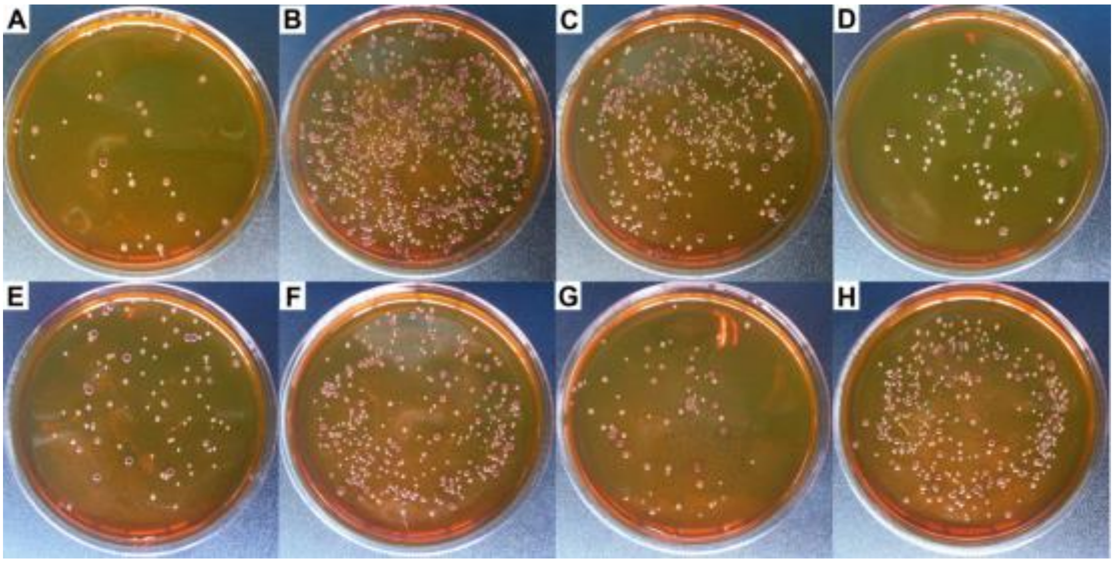

Fig. S4. The formation of *Enterobacter* colonies was formatted on Eosin-Methylene Blue Agar plates. A-H represents different exposure groups (control; DSS; ZnONPs 0.5 + DSS; ZnONPs 5 + DSS; ZnONPs 50 + DSS; ZnONPs 50 + 5-ASA + DSS; 5-ASA+DSS; ZnOMPs 50 + DSS).

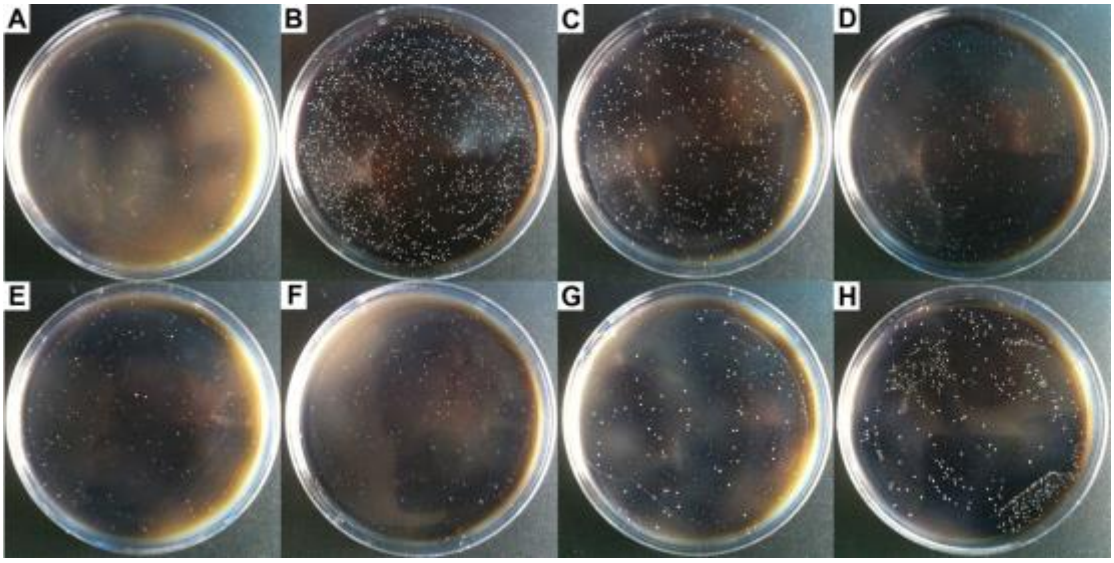

Fig. S5. The formation of *Enterococcus* colonies was formatted on Enterococcus Agar plates. A-H represents different exposure groups (control; DSS; ZnONPs 0.5 + DSS; ZnONPs 5 + DSS; ZnONPs 50 + DSS; ZnONPs 50 + 5-ASA + DSS; 5-ASA+DSS; ZnOMPs 50 + DSS).

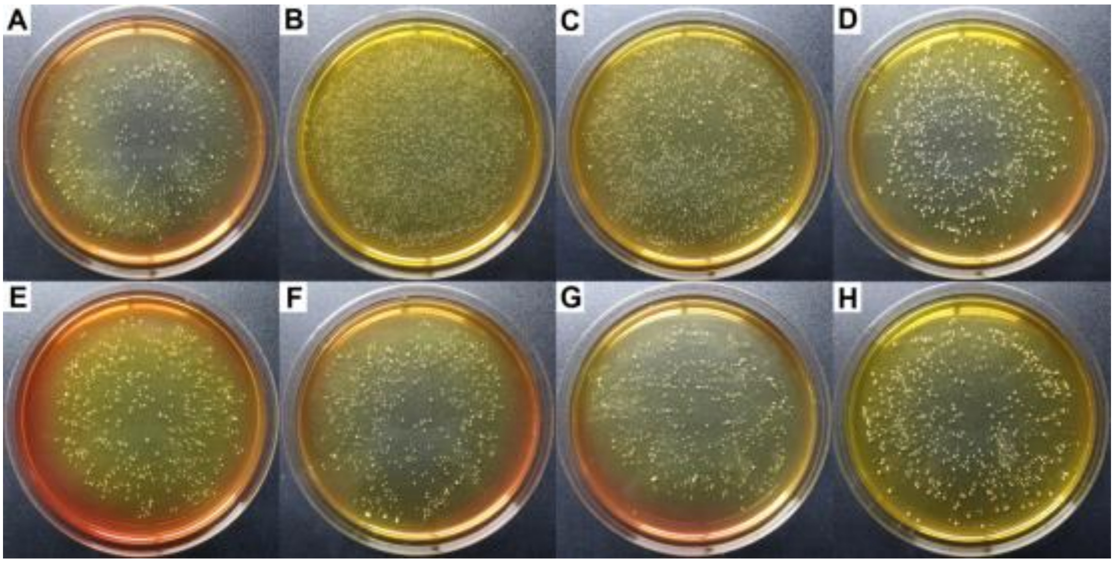

Fig. S6. The formation of *Staphylococcus aureus* colonies was formatted on Mannitol Salt Agar plates. A-H represents different exposure groups (control; DSS; ZnONPs 0.5 + DSS; ZnONPs 5 + DSS; ZnONPs 50 + DSS; ZnONPs 50 + 5-ASA + DSS; 5-ASA+DSS; ZnOMPs 50 + DSS).

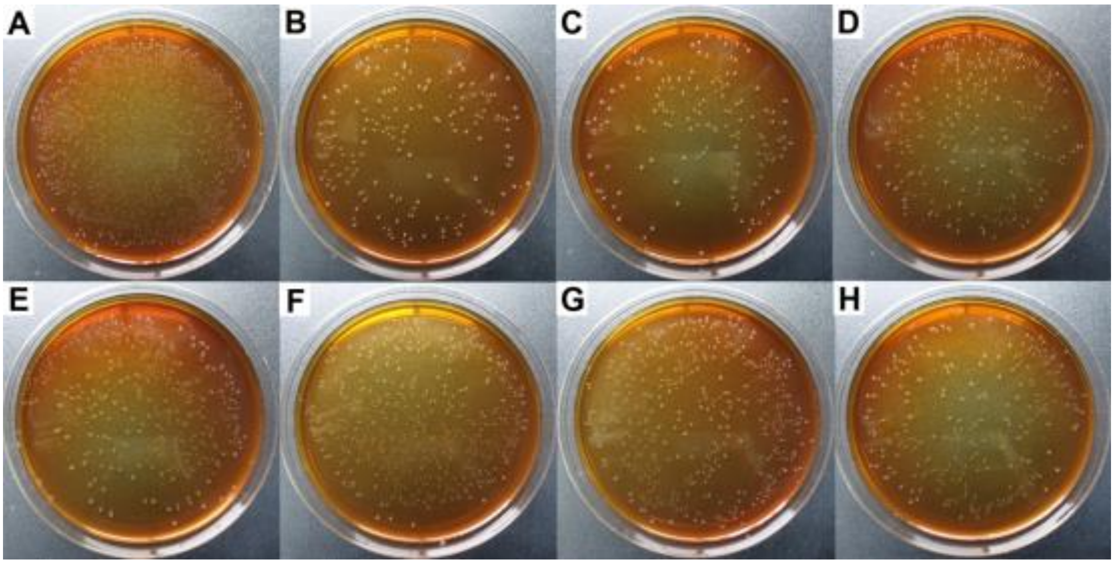

Fig. S7. The formation of *Lactobacillus* colonies was formatted on Lactobacillus Selective Agar plates. A-H represents different exposure groups (control; DSS; ZnONPs 0.5 + DSS; ZnONPs 5 + DSS; ZnONPs 50 + DSS; ZnONPs 50 + 5-ASA + DSS; 5-ASA+DSS; ZnOMPs 50 + DSS).

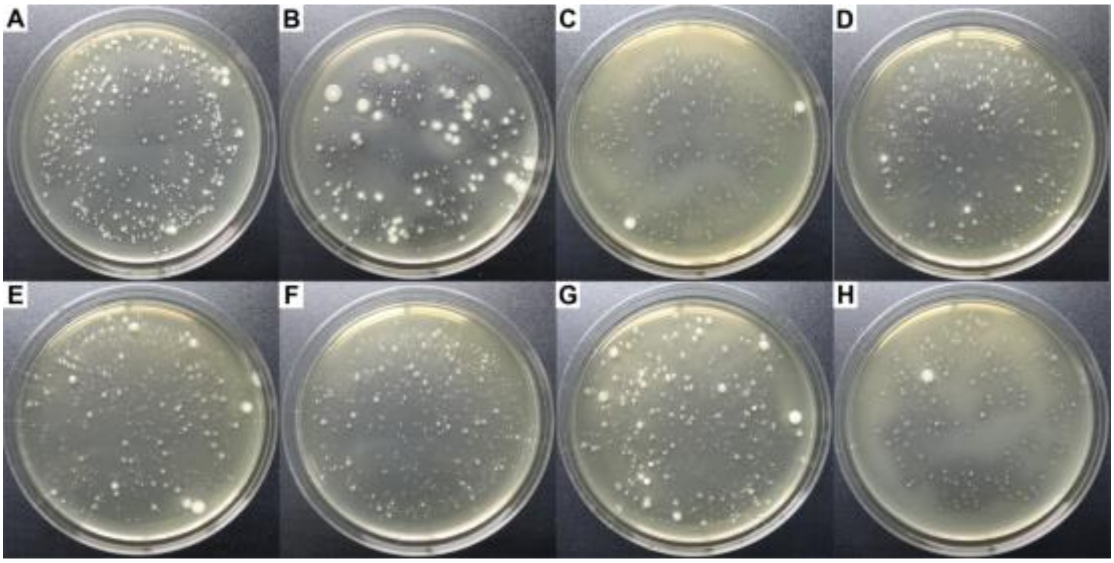

Fig. S8. The formation of *Bifidobacterium* colonies was formatted on TPY Agar plates. A-H represents different exposure groups (control; DSS, ZnONPs 0.5 + DSS; ZnONPs 5 + DSS; ZnONPs 50 + DSS; ZnONPs 50 + 5-ASA + DSS; 5-ASA+DSS; ZnOMPs 50 + DSS).

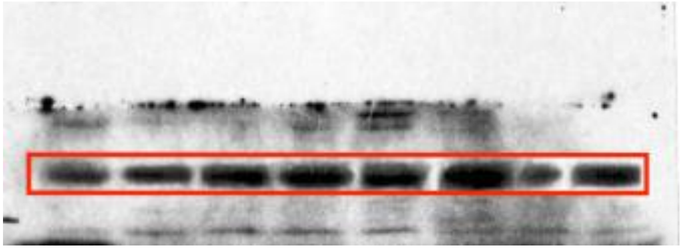

Nrf2

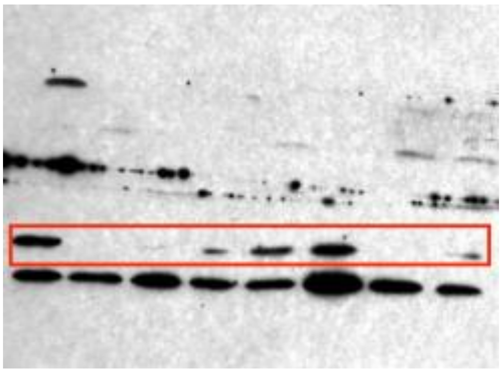

NQO-1

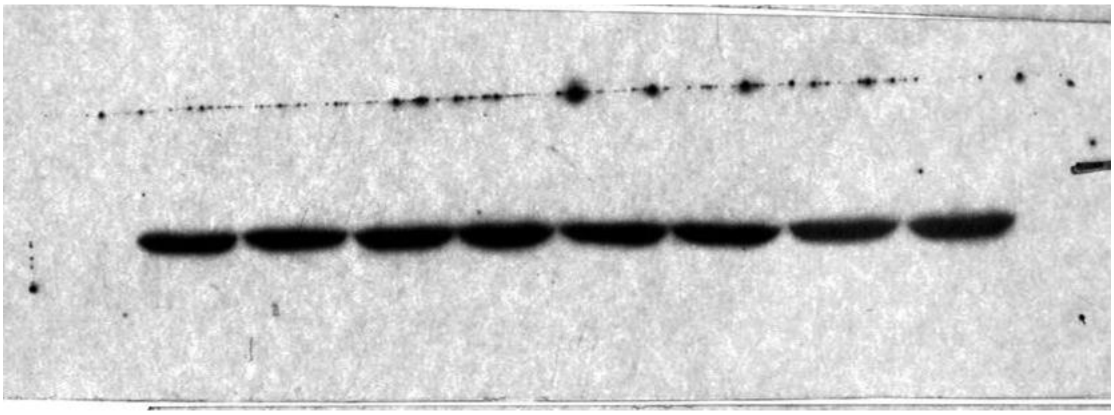

β-actin

Fig. S9. Full-length gels/blots of Figure 7.
